# Supplementary material for: Fast simulation for multi-photon, atomic-ensemble quantum model of linear optical systems addressing the curse of dimensionality
Source: Sci Rep. 2024 Feb 8;14:3208. doi: 10.1038/s41598-024-53246-2 (PMC10853269; doi:10.1038/s41598-024-53246-2)
Supplement: Supplementary file 1 — Supplementary Information. [file 41598_2024_53246_MOESM1_ESM.pdf]

Supplementary Information of "Fast simulation for multi-photon, atomic-ensemble quantum model of linear optical systems addressing the curse of dimensionality"

Junpei Oba,[1,*] Seiji Kajita,[1,†] and Akihito Soeda[2,‡]

[1] Toyota Central R&D Labs., Inc., 41-1, Yokomichi, Nagakute, Aichi 480-1192, Japan.
[2] Principles of Informatics Research Division, National Institute of Informatics, 2-1-2 Hitotsubashi, Chiyoda-ku, Tokyo 101-8430, Japan

I. HAMILTONIAN OF SINGLE-PHOTON SYSTEM

In this section, we transform a Hamiltonian of the one-photon system proposed in a previous study [1] in a form suitable for the main text. According to Ref. [1], the one-photon Hamiltonian that involves N_A two-level atoms is defined by

$$
\begin{aligned}
\hat{h} &= \hat{h}_F + \hat{h}_A + \hat{h}_I \\
\hat{h}_F &= \sum_{\mathbf{k}} \omega_{\mathbf{k}} \hat{a}^{\dagger}_{\mathbf{k}} \hat{a}_{\mathbf{k}} \\
\hat{h}_A &= \sum_{j=1}^{N_A} \omega_j \hat{\sigma}_j .
\end{aligned}
$$

The Hamiltonian of the free propagation of the photon, $\hat{h}_F$, consists of an annihilation operator $\hat{a}_{\mathbf{k}}$ for one photon that has a wave-number vector $\mathbf{k}$. That of the two-level atoms, $\hat{h}_A$, is composed of the Pauli z operator $\hat{\sigma}_j$ of the j-th two-level atom. The parameters $\omega_{\mathbf{k}}$ and ω_j indicate eigen energies of the photon and two-level atom, respectively. The $\hat{h}_I$ represents interaction between the photon and two-level atoms.

A quantum state of the corresponding system is written by

$$
|\phi\rangle = \sum_{\mathbf{k}} c(\mathbf{k}) |1_{\mathbf{k}}\rangle + \sum_{j=1}^{N_A} c_j |1_j\rangle , \tag{1}
$$

where $c(\mathbf{k})$ and c_j are coefficients of the probability amplitudes. When operating $\hat{h}_A$ to the state, we obtain

$$
\begin{aligned}
\hat{h}_A |\phi\rangle &= -\sum_j \omega_j \sum_{\mathbf{k}} c(\mathbf{k}) |1_{\mathbf{k}}\rangle + \sum_{j,j'} (\delta_{j,j'} - \delta_{j\neq j'}) \omega_j c_{j'} |1_{j'}\rangle \\
&= -N_A \omega \sum_{\mathbf{k}} c(\mathbf{k}) |1_{\mathbf{k}}\rangle + \sum_{j,j'} (2\delta_{j,j'} - 1) \omega_j c_{j'} |1_{j'}\rangle \\
&= -N_A \omega \sum_{\mathbf{k}} c(\mathbf{k}) |1_{\mathbf{k}}\rangle + 2 \sum_j c_j \omega_j |1_j\rangle - N_A \omega \sum_j c_j |1_{j'}\rangle \\
&= -N_A \omega |\phi\rangle + 2 \sum_j c_j \omega_j |1_j\rangle \\
&\equiv (-N_A \omega + \hat{h}_a) |\phi\rangle ,
\end{aligned}
$$

where $\omega = \sum_j \omega_j / N_A$, and we defined $\hat{h}_a$ as

$$
\hat{h}_a = \sum_{j=1}^{N_A} 2\omega_j \hat{a}^{\dagger}_j \hat{a}_j , \tag{2}
$$

* junpei-oba@mosk.tytlabs.co.jp
† fine-controller@mosk.tytlabs.co.jp
‡ soeda@nii.ac.jp

Therefore, the Hamiltonian becomes

$$\hat{h} \;=\; \hat{h}_0 + \hat{h}_I - N_A\omega$$

where $\hat{h}_0 = \hat{h}_F + \hat{h}_a$. Because the constant energy $-N_A\omega$ does not influence the time evolution, we omit this term when displaying the Hamiltonian in the main text.

The Hamiltonian $\hat{h}_I$ that describes the dipole-dipole interaction between the two-level atoms and photons as

$$\begin{aligned} \hat{h}_I \;&=\; \sum_{j,\mathbf{k}} (g(j,\mathbf{k})\hat{a}_j^\dagger \hat{a}_\mathbf{k} + g^*(j,\mathbf{k})\hat{a}_\mathbf{k}^\dagger \hat{a}_j) \qquad (3) \\ g(j,\mathbf{k}) \;&=\; -\frac{\mathrm{i}}{2L}\sqrt{\omega_\mathbf{k}} D_j e^{\mathrm{i}\mathbf{k}\cdot\mathbf{r}_j}. \end{aligned}$$

We set the frequencies of the two-level atoms and photon to be in resonance in simulation, thus $g(j,\mathbf{k})$ is approximated as

$$g(j,\mathbf{k}) = -\frac{\mathrm{i}}{\sqrt{2}L}\sqrt{\omega_j} D_j e^{\mathrm{i}\mathbf{k}\cdot\mathbf{r}_j}. \qquad (4)$$

[1] Havukainen, M., Drobný, G., Stenholm, S., & Bužek, V. Quantum simulations of optical systems. *Journal of Modern Optics* **46**, 1343-1367 (1999).
